# Haloperidol Abrogates Matrix Metalloproteinase-9 Expression by Inhibition of NF-*κ*B Activation in Stimulated Human Monocytic Cells

**DOI:** 10.1155/2018/9541459

**Published:** 2018-04-12

**Authors:** Yueh-Lun Lee, Che-Jen Hsiao, Fan-Li Lin, Jing-Shiun Jan, Yung-Chen Chou, Yen-Yu Lin, Chih-Kuang Chen, Kwok-Keung Lam, George Hsiao

**Affiliations:** ^1^Department of Microbiology and Immunology, School of Medicine, College of Medicine, Taipei Medical University, Taipei, Taiwan; ^2^Laboratory of Neural Repair, Department of Medical Research, China Medical University Hospital, Taichung, Taiwan; ^3^Graduate Institute of Medical Sciences and Department of Pharmacology, College of Medicine, Taipei Medical University, Taipei, Taiwan; ^4^Graduate Institute of Clinical Medicine, College of Medicine, Taipei Medical University, Taipei, Taiwan; ^5^Department of Physical Medicine and Rehabilitation, Chang Gung Memorial Hospital, Taoyuan, Taiwan; ^6^Department of Anesthesiology, Catholic Mercy Hospital, Hsinchu, Taiwan; ^7^Ph.D. Program in Biotechnology Research and Development, College of Pharmacy, Taipei Medical University, Taipei, Taiwan

## Abstract

Much evidence has indicated that matrix metalloproteinases (MMPs) participate in the progression of neuroinflammatory disorders. The present study was undertaken to investigate the inhibitory effect and mechanism of the antipsychotic haloperidol on MMP activation in the stimulated THP-1 monocytic cells. Haloperidol exerted a strong inhibition on tumor necrosis factor- (TNF-) *α*-induced MMP-9 gelatinolysis of THP-1 cells. A concentration-dependent inhibitory effect of haloperidol was observed in TNF-*α*-induced protein and mRNA expression of MMP-9. On the other hand, haloperidol slightly affected cell viability and tissue inhibition of metalloproteinase-1 levels. It significantly inhibited the degradation of inhibitor-*κ*B-*α* (I*κ*B*α*) in activated cells. Moreover, it suppressed activated nuclear factor-*κ*B (NF-*κ*B) detected by a mobility shift assay, NF-*κ*B reporter gene, and chromatin immunoprecipitation analyses. Consistent with NF-*κ*B inhibition, haloperidol exerted a strong inhibition of lipopolysaccharide- (LPS-) induced MMP-9 gelatinolysis but not of transforming growth factor-*β*1-induced MMP-2. In *in vivo* studies, administration of haloperidol significantly attenuated LPS-induced intracerebral MMP-9 activation of the brain homogenate and the in situ in C57BL/6 mice. In conclusion, the selective anti-MMP-9 activation of haloperidol could possibly involve the inhibition of the NF-*κ*B signal pathway. Hence, it was found that haloperidol treatment may represent a bystander of anti-MMP actions for its conventional psychotherapy.

## 1. Introduction

Neurogenic or cerebromicrovascular inflammation has been postulated as a crucial etiological factor in the onset and progression of schizophrenia [1]. Consistently, the proinflammatory tumor necrosis factor- (TNF-) *α* significantly increases in the prefrontal cortex of schizophrenic subjects [2]. In addition, chronically activated monocytes/macrophages and T-lymphocytes were also proposed as being fundamental mediators of schizophrenia [3]. Therefore, anti-inflammatory therapy has produced improvement in some schizophrenic patients [4].

The inflammation-related matrix-degrading enzymes matrix metalloproteinases (MMPs) are neutral zinc-dependent endoproteinases encoded by a multigene family that are capable of degrading components of the extracellular matrix (ECM) or some specific bioactive proteins [5]. The neurophysiological functions of MMPs are also critical to synaptic plasticity and spatial learning [6]. However, it has also been suggested that MMPs play important roles in the pathogenesis of chronic neurodegenerative disorders [7] and schizophrenia [8]. Recently, it was found that the changes of MMP-9 levels in the excitatory synapse and morphology of the dendritic spine could be involved in the pathophysiology of schizophrenia [9]. In particular, the monocyte/macrophage lineage plays a crucial role in the inflammatory remodeling by releasing MMPs [10]. Since TNF-*α* is a crucial inducer of MMP-9 expression, the Toll-like receptor-4 activator lipopolysaccharide (LPS) could also upregulate TNF-*α* to amplify the inflammatory responses by increasing further MMP-9 production [11]. Secreted MMP-9 targets not only ECM substrates but also nonmatrix substrates such as *α*B-crystalline [12] and even exacerbates neurotoxicity [13]. The elevated expression or activation of MMP-9 was clinically found to be associated with brain edema [14] and familial amyloidotic polyneuropathy [15]. Treatment with MMP inhibitors and MMP-9 knockout in mice substantially reduced brain edema and neuronal death [16, 17].

Haloperidol is believed to principally act as a potent dopaminergic antagonist and is one of the most widely prescribed conventional antipsychotic drugs [18]. On the other hand, chronic administration of haloperidol was shown to inhibit the NF-*κ*B p50 expression in a specific brain region [19]. Interestingly, the activity of protein phosphatase 2A in the frontal cortex was regulated by the acute action of haloperidol [20]. Furthermore, haloperidol can also normalize increased release of proinflammatory cytokines such as interleukin-1*β* and TNF-*α* from activated monocytes in schizophrenia [21]. This antipsychotic agent also exerted an antineuroinflammatory activity on inhibition of the secretion of S100B following interleukin-6 stimulation in brain C6 glioma cells [22].

Therefore, the hypothesis is that haloperidol may exert its inhibitory effects on neuroinflammation and abnormal remodeling, particularly on MMP expression in activated inflammatory cells. In the present study, we clarified the inhibitory mechanisms of haloperidol on TNF-*α*-induced MMP-9 expression in human monocytic cells. We also investigated its effect on the activation of MMP-9 in LPS-induced neuroinflammation *in vivo*.

## 2. Materials and Methods

### 2.1. Chemicals and Reagents

Haloperidol, chlorpromazine, MG-132, pyrrolidine dithiocarbamate (PDTC), aprotinin, phenylmethylsulfonyl fluoride (PMSF), *β*-mercaptoethanol, brilliant blue G-colloidal concentrate, Brij 35 solution, diethyl pyrocarbonate (DEPC), dithiothreitol (DTT), N-(2-hydroxyethyl)piperazine-N′-(2-ethanesulfonic acid) (HEPES), leupeptin, lipopolysaccharide (LPS; *Escherichia coli*, serotype 0127: B8), parthenolide, and 3-(4,5-dimethylthiazol-2-yl)-2,5-diphenyltetrazolium bromide (MTT) were purchased from Sigma-Aldrich (St. Louis, MO). 2,2,5,7,8-pentamethyl-6-hydroxychroman (PMC) was obtained from Wako Pure Chemical (Osaka, Japan). SP600125 was purchased from Tocris (Ellisville, MO). Recombinant human transforming growth factor-*β*1 (TGF-*β*1) was obtained from PeproTech (Rocky Hill, NJ). The murine anti-MMP-9 monoclonal antibody (mAb) was purchased from Neomarkers (Fremont, CA). Anti-mouse or anti-rabbit immunoglobulin G (IgG) linked to horseradish peroxidase antibodies (Abs) and the Western blotting detection system (ECL^+^) were purchased from Amersham Biosciences (Buckinghamshire, UK). Tissue culture media, supplements, and fetal bovine serum (FBS) were purchased from Invitrogen (Carlsbad, CA). All other reagents used were of the highest grade available.

### 2.2. Cell Cultivation

THP-1 cells, a human monocytic cell line, were obtained from American Type Cell Culture Collection (Manassas, VA) and grown in RPMI 1640 supplemented with 10% heat-inactivated FBS, 20 mM HEPES, 2 mM L-glutamine, 100 units/ml penicillin G, and 100 *μ*g/ml streptomycin in a humidified 37°C incubator with 5% CO_2_. Before experiments, cells were centrifuged at 75 ×g for 5 minutes. The cell pellet was resuspended to a cell concentration of 1 × 10^6^ (cells/ml) in the 0.5% heat-inactivated FBS supplemented RPMI 1640 medium as described [23].

### 2.3. Gelatin Zymography

The MMP-mediated gelatinolytic capacity was evaluated as described [24]. Briefly, THP-1 cells (1 × 10^6^ cells/ml; 500 *μ*l/well) were dispensed onto 24-well plates (Costar, Cambridge, MA). The cells were pretreated with vehicle (DMSO) or various concentrations of haloperidol (0.5–20 *μ*M) for 15 min followed by the addition of TNF-*α* (10 ng/ml) for 24 h. In other stimulating conditions, the concentration of TGF-*β*1 or LPS that was used was 50 ng/ml, referred to by the preliminary concentration-response curve. The specific gelatinolytic bands were analyzed using the same digital imaging system and analytic software as previously described.

### 2.4. Cell Viability Assay

The viability of THP-1 cell after 24 h of continuous exposure to haloperidol (10–40 *μ*M) was evaluated by a spectrophotometric assay based on the ability of mitochondria to reduce the MTT in viable cells as described previously [24]. The percentage cell viability was calculated as (absorbance of treated cells/absorbance of control cells) × 100%.

### 2.5. Enzyme-Linked Immunosorbent Assay (ELISA) for TIMP-1

The amount of secreted TIMP-1 protein was quantified using the highly specific Biotrak Matrix TIMP-1 ELISA system (Amersham). TIMP-1 levels were corrected according to the cell number, and data are shown as nanograms per 10^6^ cells.

### 2.6. Western Blot Analyses

Briefly, THP-1 cells (1 × 10^6^ cells/ml; 3.0 ml/well) were cultured in the wells of a 6-well plate (Costar). The cells were pretreated with haloperidol (0.5–20 *μ*M) for 15 min followed by the addition of TNF-*α* (10 ng/ml) for the indicated times. After cell lysis and gel electrophoresis, transfer membranes were incubated with either an anti-MMP-9 mAb (1 : 350; Neomarkers), anti-phospho-JNK (Thr 183/Tyr 185), and total-JNK mAb (1 : 2000; Cell Signaling Technology, Danvers, MA) or a rabbit anti-human I*κ*B*α* Ab (1 : 3000; Santa Cruz Biotechnology, Santa Cruz, CA). Densitometric analysis of specific bands was performed as previously described [25].

### 2.7. Isolation of Total RNA and Reverse-Transcription Polymerase Chain Reaction (RT-PCR)

Isolation of total RNA and RT-PCR was performed as previously described [26]. The primers used to target MMP-9 were 5′-CGTGGAGAGTCGAAATCTCTG-3′ (sense) and 5′-CCAAACTGGATGACGATGTCT-3′ (antisense). The glyceraldehyde-3-phosphate dehydrogenase (GAPDH) primers were 5′-CCACCCATGGCAAATTCCATGGCA-3′ (sense) and 5′-TCAAGACGGCAGGTCAGGTCCACC-3′ (antisense). Densitometric analysis of the specific bands of the PCR products was performed as previously described.

### 2.8. Electrophoretic Mobility Shift Assay (EMSA)

The electrophoretic mobility shift assay was performed as previously described [24]. The nuclear extracts of manipulated THP-1 cells were prepared using NE-PER™ nuclear and cytoplasmic extraction reagents (Pierce, Rockford, IL). The biotin-labeled NF-*κ*B probe consisted of the consensus oligonucleotide (5′-AGTTGAGGGGACTTTCCCAGG-3′). EMSAs were performed with the LightShift Chemiluminescent EMSA Kit (Pierce) with minor modifications. Supershift assays were used to confirm the identity of the p65-NF-*κ*B-binding complex.

### 2.9. Transfection and Luciferase Assay

The NF-*κ*B-dependent reporter expression was evaluated as previously described [26]. THP-1 cells were transfected with the specific NF-*κ*B reporter plasmid using the Effectene Transfection Reagent (Qiagen, Valencia, CA). After 12 h of transfection, cells were incubated and starved for an additional 1 h and then stimulated with TNF-*α* (10 ng/ml) for the indicated times. The luciferase activity was measured by the Dual-Luciferase Reporter Assay System (Promega). The luciferase activity was measured and normalized to the activity of Renilla luciferase as the internal control.

### 2.10. Chromatin Immunoprecipitation (ChIP) Assay

The ChIP assays (Upstate, Charlottesville, VA) were performed as previously described [26]. After fixation and sonication, the cellular supernatants were subjected to immunobinding with anti-p65 NF-*κ*B antibody (Santa Cruz) at 4°C with rotation overnight, followed by incubation with protein A agarose for 1 h. Following eluates of the specific protein and DNA complexes, the crosslink was reversed by heating to 65°C for 5 h. The DNA was extracted with EasyPure PCR/Gel extraction kit (Bioman, Taipei, Taiwan). The purified DNA was analyzed for the presence of the MMP-9 upstream promoter region by PCR using the following primers: forward, 5′-CACTTCAAAGTGGTAAGA-3′, and reverse, 5′-GAAAGTGATGGAAGACTCC-3′.

### 2.11. The Intrastriatal MMP-9 Activation by Microinjection of LPS in C57BL/6 Mice

The animal studies were approved by the Taipei Medical University Institutional Animal Care and Use Committee (LAC-95-0145). All animal experiments and care were performed according to the Guide for the Care and Use of Laboratory Animals (Washington, DC: National Academy Press, 1996). Male C57BL/6 mice (23–28 g) were anesthetized with chloral hydrate (400 mg/kg) and underwent manipulation by a stereotaxic frame (Stoelting, Wood Dale, IL). The cranium of the mouse was drilled and injected with normal saline (NS) or LPS solution unilaterally into the right caudate/putamen region, using the stereotactic coordinates (0.3 mm anterior and 2.0 mm lateral of the bregma, 3.8 mm in depth). Each mouse received a 3 *μ*l injection (equivalent to 4 *μ*g LPS) by a 26s-gauge gastight syringe (10 *μ*l, Hamilton; Reno, NV) with the microinfusion syringe pump (Singa®, Diagnostic and Research Instruments Inc., Taipei, Taiwan) over 2 min; the needle was retained in place for an additional 5 min to prevent any reflux. To examine the effects of haloperidol on MMP-9 activation, haloperidol (5 mg/kg) or its solvent (lactate solution, 10 mg/ml) were twice administered 60 min before and 25 min after LPS treatment. After 8 hours LPS treatment, animals were anesthetized and perfused transcardially with 4°C NS for 15 min. The fresh and cooled brains were sectioned coronally into four sequential parts from the frontal to the occipital lobe by using Adult Mouse Brain Matrices (Model 0530, Vibratome, St. Louis, MO). The right and left hemispheres of the part with the injected site were separately collected. Each brain tissue sample was homogenized in 200 *μ*l of ice-cold 50 mM Tris-HCl (pH 7.4) buffer containing 150 mM NaCl, 0.2% Triton X-100, and complete EDTA-free protease inhibitor cocktail tablet solution (×1) (Roche, Mannheim, Germany). The homogenates were centrifuged at high speed (13,000) for 30 min. The resulting supernatants were stored at −70°C. Each brain supernatant (10 *μ*g of protein) was applied to gelatin-substrate zymography as previously described.

### 2.12. In Situ Zymography

Gelatinolytic activity of MMPs was localized on frozen tissue sections by in situ zymography [27]. Briefly, fresh brains were removed without fixation, then immersed and frozen in ornithine carbamoyltransferase (OCT) compound (Tissue-Tek, Torrance, CA) by dry ice. The brain in the OCT block was cut into 15 *μ*m sections using a cryostat (Thermo-Shandon Cryotome E; Thermo Electron Corporation, Waltham, MA). The sections were incubated with 0.05 M Tris-HCl, 0.15 M NaCl, 5 mM CaCl_2_, and 0.2 mM NaN_3_, pH 7.6, also containing 40 *μ*g/ml of DQ gelatin (EnzChek; Molecular Probes, Eugene, OR) in a moist chamber at 37°C for 16 h. At the end of the incubation, gelatinolytic activities of MMPs were localized and visualized by a fluorescence microscope (ECLIPSE 80i, Nikon) with Evolution MP 5.0 Cooled Color Camera Kit and Image-Pro® Express (v 6.0, Media Cybernetics, MD).

### 2.13. Statistical Analysis

The experimental results are expressed as the mean ± SEM and are accompanied by the number of observations. Data were assessed by Student's unpaired *t*-test unless specifically mentioned. A *p* value of less than 0.05 was considered statistically significant.

## 3. Results

### 3.1. Effects of Haloperidol on MMP-9-Induced Gelatinolysis and Viability in TNF-*α*-Stimulated THP-1 Cells

**Figure 1:**
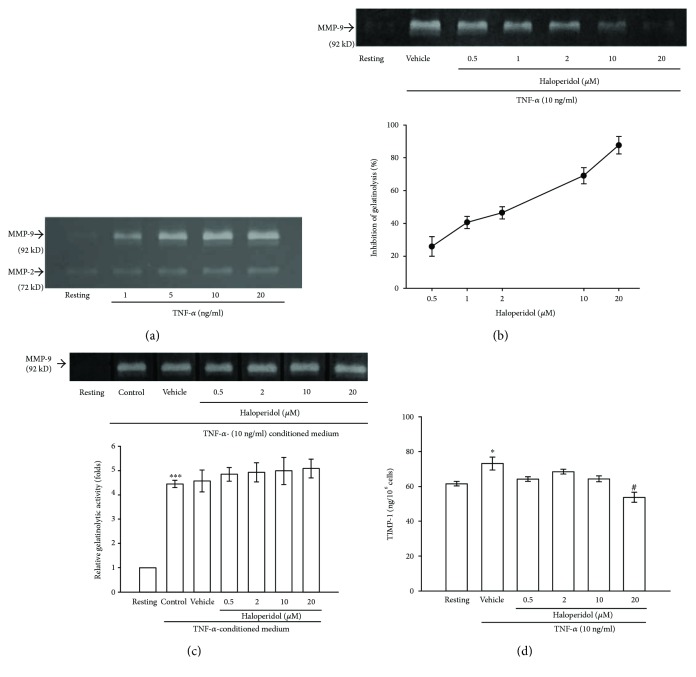
Effect of haloperidol on tumor necrosis factor- (TNF-) *α*-induced gelatinolytic activity of matrix metalloproteinase- (MMP-) 9, TIMP-1 contents in THP-1 cells, and native MMP-9 enzymatic activity. (a) THP-1 cells (1 × 10^6^ cells/ml) were dispensed on 24-well plates and stimulated with various concentrations of TNF-*α* for 24 h. (b) THP-1 cells were pretreated with haloperidol (0.5, 1, 2, 10, and 20 *μ*M) or vehicle (DMSO) for 15 min before treatment with TNF-*α* (10 ng/ml). Cells were not treated with the stimulator (lane 1, resting). The cell-free conditioned medium was obtained and assayed for MMP-9-mediated gelatinolysis by gelatin zymography, as detailed in Methods. The concentration-inhibition curve of haloperidol on MMP-9-mediated gelatinolysis was performed as below. The percent inhibition was presented as the mean ± SEM of four independent experiments. (c) In a representative zymographic analysis of intact MMP-9 enzymatic activity, TNF-*α*- (10 ng/ml) conditioned medium was obtained. Vehicle or haloperidol (0.5, 2, 10, and 20 *μ*M) was added into the incubation buffer as described in Methods. Bottom: densitometric analysis of bands for enzymatic activity normalized to the resting group. Data are the representative example of four experiments. ^∗∗∗^*p* < 0.001, compared to the resting group. (d) THP-1 cells (1 × 10^6^ cells/ml) were pretreated with vehicle or haloperidol (0.5, 2, 10, and 20 *μ*M) for 15 min before treatment with TNF-*α* (10 ng/ml) for 24 h. Cell-free supernatants were assayed for TIMP-1 contents by ELISA. Data of TIMP-1 content (ng/10^6^ cells) are presented as the mean ± SEM of three independent experiments. ^∗^*p* < 0.05, compared to the resting group; ^#^*p* < 0.05, compared to the vehicle under stimulation.

Within 24 h, TNF-*α* (1, 5, 10, and 20 ng/ml) induced a concentration-dependent increase in latent MMP-9- (92 kD) mediated gelatinolysis in the culture medium of THP-1 cells by 1.9 ± 0.4-, 3.0 ± 0.3-, 4.0 ± 0.1-, and 4.2 ± 0.2-folds compared to resting condition, respectively (*n* = 4, Figure 1(a)). Therefore, cells were treated with a submaximal concentration of TNF-*α* of 10 ng/ml for 24 h in the following experiments. After pretreating cells with various concentrations of haloperidol (0.5, 1, 2, 10, and 20 *μ*M) for 15 min followed by the addition of TNF-*α* (10 ng/ml), we found that haloperidol concentration dependently inhibited MMP-9-mediated gelatinolysis stimulated by TNF-*α* (Figure 1(b)). At 0.5, 1, 2, 10, and 20 *μ*M, haloperidol inhibited this gelatinolytic reaction by about 25.6 ± 6.1, 40.4 ± 3.7, 46.3 ± 3.7, 69.0 ± 4.9, and 87.7 ± 5.3%, respectively. The IC_50_ value of haloperidol on the MMP-9 activation was about 3.7 ± 0.7 *μ*M (*n* = 4). The active form of MMP-9 (below 92 kD) was also attenuated by haloperidol in a concentration-dependent manner (Figure 1(b)). According to the MTT assays, neither haloperidol (10 and 20 *μ*M) nor the solvent control (0.1% DMSO) strongly affected the cellular viability. Viability was approximately 95% and 78% with haloperidol at 10 and 20 *μ*M, respectively. To confirm the identity of the MMPs, identical gels were incubated in the reacting buffer containing EDTA (20 mM), and the treatments completely abolished gelatinolysis (data not shown).

### 3.2. Effects of Haloperidol on Native MMP-9 Enzymatic Activity and TIMP-1 Content

The native enzymatic inhibition of monocytic MMP-9 was studied by analyzing aliquots of MMP-9-containing medium by gelatin zymography developed in the presence of increasing concentrations of haloperidol. As expected, latent MMP-9 enzymes from TNF-*α*-induced THP-1 cells caused a strong 4.5 ± 0.2-fold increase in gelatinolysis compared to the control value (*n* = 4, Figure 1(c)). Treatment of the MMP-9 enzyme with concentrations of haloperidol (0.5–20 *μ*M) resulted in no significant effect on gelatinolysis (Figure 1(c)), whereas treatment with 1,10-phenanthroline (1 mM), as a broad MMP inhibitor, markedly abolished gelatinolysis by over 95% (data not shown). Therefore, haloperidol did not interfere with the native enzymatic activity of MMP-9 under these experimental conditions.

On the other hand, the concentration of secreted TIMP-1 was significantly upregulated from 61.6 ± 1.3 to 73.2 ± 3.8 ng/10^6^ cells after the addition of TNF-*α* (10 ng/ml) for 24 h (Figure 1(d)). After pretreatment with various concentrations of haloperidol (0.5–20 *μ*M) followed by stimulation with TNF-*α* (10 ng/ml), we found that haloperidol only at the high concentration of 20 *μ*M partially inhibited the production of TIMP-1 with a value of 53.8 ± 2.9 ng/10^6^ cells (*p* < 0.05, Figure 1(d)). These results demonstrate that inhibition of gelatinolytic activity by haloperidol was due to neither the reduced enzyme catalytic activity of MMP-9 nor the TIMP-1 upregulation in THP-1 cells.

### 3.3. Effects of Haloperidol on TNF-*α*-Stimulated Expression of MMP-9 Protein and mRNA

**Figure 2:**
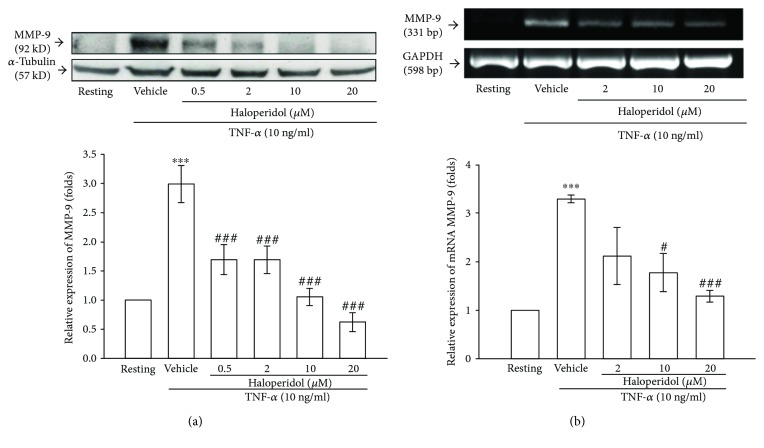
Effects of haloperidol on TNF-*α*-induced expression of MMP-9 and its mRNA. (a) Cell lysates were obtained and analyzed for MMP-9 protein expression by Western blotting. THP-1 cells (1 × 10^6^ cells/ml) were pretreated with haloperidol (0.5, 2, 10, and 20 *μ*M) or vehicle (DMSO) for 15 min before treatment with TNF-*α* (10 ng/ml) for 24 h. Cells were not treated with the stimulator (lane 1, resting). Bottom: densitometric analyses of bands for MMP-9, relative to *α*-tubulin normalized to the resting group. Data are representative example of five to six experiments. ^∗∗∗^*p* < 0.001, compared to the resting group; ^###^*p* < 0.001, compared to the vehicle. (b) RT-PCR analyses of MMP-9 mRNA expression in THP-1 cells. Cells were pretreated with haloperidol (2, 10, and 20 *μ*M) or vehicle for 15 min before treatment with TNF-*α* (10 ng/ml) for 6 h. Following extraction of total RNA, the relative mRNA levels of MMP-9 and GAPDH were analyzed by RT-PCR as described in Methods. GAPDH levels were used to normalize the amount of cDNA temple used in each PCR reaction. Data are representative examples of three experiments. Bottom: densitometric analyses of bands for MMP-9 mRNA, relative to GAPDH mRNA, normalized to the resting group. ^∗∗∗^*p* < 0.001, compared to the resting group; ^#^*p* < 0.05 and ^###^*p* < 0.001, compared to the vehicle under stimulation.

As shown in Figure 2(a), TNF-*α*- (10 ng/ml) induced expression of MMP-9 protein was markedly increased by 3.0 ± 0.3-fold compared to that of the resting control after 24 h of treatment (Figure 2(a), lanes 1 and 2). Pretreatment with haloperidol (0.5, 2, 10, and 20 *μ*M) for 15 min before TNF-*α* treatment revealed that it markedly inhibited the expression of MMP-9 protein in a concentration-dependent manner (Figure 2(a), lanes 3 to 6). At a higher concentration of 10 *μ*M, haloperidol inhibited the expression of MMP-9 protein by about 95% (*n* = 4) (Figure 2(a)).

Furthermore, TNF-*α* (10 ng/ml) markedly stimulated a 3.3 ± 0.1-fold increase in MMP-9 mRNA in THP-1 cells compared to the resting control (Figure 2(b), lanes 1 and 2). Pretreatment with various concentrations of haloperidol (2, 10, and 20 *μ*M) for 15 min markedly inhibited the expression of MMP-9 mRNA stimulated by TNF-*α* by about 52%, 65%, and 87% (*n* = 4), respectively (Figure 2(b), lanes 3 to 5).

### 3.4. Effects of Haloperidol on I*κ*B*α* Degradation and JNK Activation

**Figure 3:**
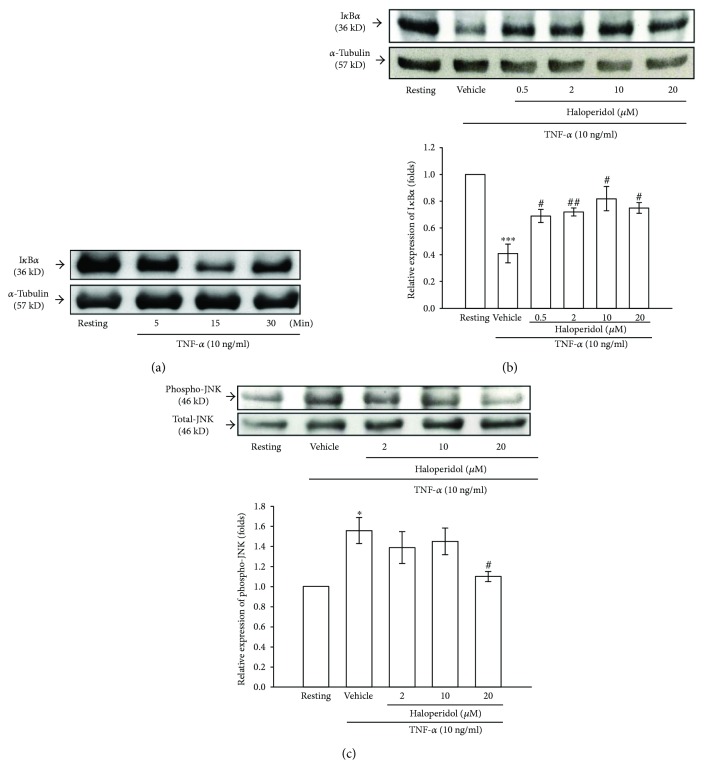
Effect of haloperidol on the degradation of I*κ*B*α* and JNK activation in stimulated THP-1 cells. (a) Western blot analyses demonstrating the time course on TNF-*α*-induced degradation of I*κ*B*α* in THP-1 cells. THP-1 cells (1 × 10^6^ cells/ml) were dispensed on 6-well plates and treated with TNF-*α* (10 ng/ml) for the indicated times (5, 15, and 30 min). Cells were not stimulated for 30 min (lane 1, Resting). (b) Degradation of I*κ*B*α* by TNF-*α* stimulation for 15 min was evaluated after pretreatment with haloperidol (0.5, 2, 10, and 20 *μ*M) or vehicle. Bottom: densitometric analyses of bands were normalized to the resting groups. Data are representative examples of three to four experiments. ^∗∗∗^*p* < 0.001, compared to the resting group; ^#^*p* < 0.05 and ^##^*p* < 0.01, compared to the vehicle under stimulation. (c) The manipulated cells were obtained and analyzed for phosphorylated (Phospho-) and total (Total-) JNK protein levels. Bottom: densitometric analysis of bands for phospho-JNK relative to total-JNK, normalized to the resting group. Data are representative examples of three experiments. ^∗^*p* < 0.05, compared to the resting group; ^#^*p* < 0.05, compared to the vehicle group.

To further investigate the inhibitory mechanisms of haloperidol on the reduction of MMP-9 expression in TNF-*α*-stimulated THP-1 cells, we detected the signaling molecules including I*κ*B*α* and p46 JNK MAPK. The immunoblotting analyses revealed that treatment with TNF-*α* (10 ng/ml) caused a rapid and time-dependent disappearance of the immunoreactive bands of I*κ*B*α* (Figure 3(a)). The I*κ*B*α* protein was markedly degraded within 15 min of TNF-*α* stimulation and returned to basal levels after 30 min. Haloperidol (0.5, 2, 10, and 20 *μ*M) significantly and concentration-dependently attenuated the degradation of the immunoreactive bands of I*κ*B*α* with 15 min of TNF-*α* stimulation (Figure 3(b)). Even at a lower concentration of 2 *μ*M, haloperidol could restore the I*κ*B*α* protein level by about 53%.

In addition, stimulation of cells with TNF-*α* (10 ng/ml) for 15 min resulted in significant phosphorylation of the 46 kDa protein band (JNK1). After pretreatment with various concentrations of haloperidol followed by stimulation with TNF-*α* (10 ng/ml) for 30 min, we found that haloperidol (2 and 10 *μ*M) had not significantly attenuated the phosphorylation of JNK1. It was only partially affected at a higher concentration of 20 *μ*M (Figure 3(c)).

### 3.5. Effects of Haloperidol on p65 Transactivation and the Binding of NF-*κ*B to the MMP-9 Promoter

**Figure 4:**
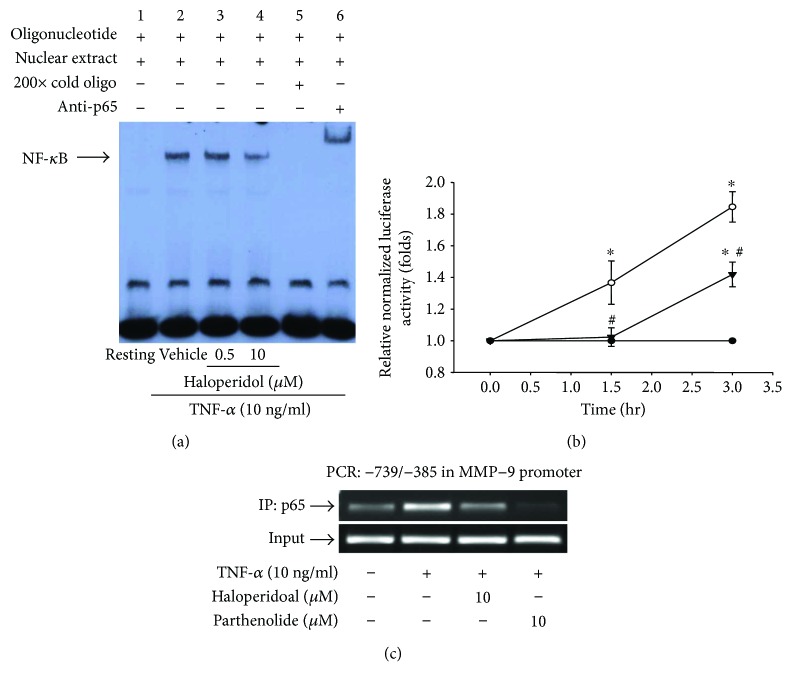
Effect of haloperidol on nuclear factor- (NF-) *κ*B transactivation and its binding to the cognate *cis*-acting element in MMP-9 promoter. (a) THP-1 cells (2 × 10^6^ cells/ml) were dispensed on 6-well plates and stimulated with TNF-*α* (10 ng/ml) for 15 min as indicated. Cells were pretreated with haloperidol (0.5 and 10 *μ*M) or vehicle (lanes 2, 5, and 6) for 15 min before treatment with TNF-*α*. Cells were not stimulated (lane 1, resting). Cellular nuclear extracts were prepared (5 *μ*g) and analyzed for NF-*κ*B activation by EMSA. Data are representative examples of three experiments. (b) At 12 h after transfection, cells were pretreated with haloperidol (10 *μ*M) (▼) or vehicle (○), then stimulated with TNF-*α* (10 ng/ml) for the indicated times. The other groups of cells were not stimulated (●). Cellular lysates were prepared for determination of luciferase activity. All luciferase activities were normalized to the internal control activity of *Renilla* luciferase. The relative changes (folds) are presented as the mean ± SEM of three independent transfections. ^∗^*p* < 0.05, compared to the resting group; ^#^*p* < 0.05, compared to the vehicle under stimulation. (c) THP-1 cells were pretreated with either vehicle, haloperidol (10 *μ*M), or parthenolide (10 *μ*M), and the cells were then stimulated with TNF-*α* for 60 min prior to the ChIP assays.

Different conditions of nuclear extracts were prepared and tested for NF-*κ*B activation as p65 transactivation by EMSA. As shown in Figure 4(a), treatment with TNF-*α* (10 ng/ml) for 15 min caused the appearance of a band of the protein-DNA complex (lanes 2) compared to the resting group (lanes 1). Haloperidol (10 *μ*M) significantly inhibited TNF-*α*-induced NF-*κ*B activation (lanes 4) with a 35% reduction compared to the stimulated control. The addition of excess unlabeled NF-*κ*B oligonucleotides (200-fold) caused the complete disappearance of the specific band (lane 5). It was also found that p65 mAb treatment shifted the band to a higher molecular mass, thus suggesting that the probe was specific for nuclear p65 (lane 6).

To further investigate the inhibitory action of haloperidol on NF-*κ*B activation in response to TNF-*α* stimulation, we first examined the effect of TNF-*α* on the transcriptional activation of NF-*κ*B in THP-1 transfected with the reporter plasmid (Clontech) containing four NF-*κ*B consensus sequences upstream of luciferase [28]. As shown in Figure 4(b), NF-*κ*B-directed promoter activity was significantly increased by 1.37 ± 0.14- and 1.85 ± 0.10-folds compared to each resting control at 1.5 and 3 h, respectively, after exposure to TNF-*α* (10 ng/ml). In transfected cells without TNF-*α* stimulation, no inducible luciferase activity was observed. We next examined the effects of haloperidol on luciferase activity in transfected THP-1 cells. Haloperidol (10 *μ*M) significantly suppressed luciferase activity by 1.02 ± 0.06- and 1.42 ± 0.08-folds compared to each resting control at 1.5 and 3 h, respectively (Figure 4(b)).

Furthermore, the interaction of NF-*κ*B with the MMP-9 gene promoter was assessed by ChIP assays. The specific PCR primers were designed to flank the NF-*κ*B site on the MMP-9 promoter for the detection of NF-*κ*B binding through the ChIP assays. It was shown that TNF-*α* significantly induced p65 binding to the NF-*κ*B site in THP-1 cells (Figure 4(c)). TNF-*α*-stimulated p65 binding was greatly attenuated in the presence of haloperidol or parthenolide (10 *μ*M). Taken together, these results confirmed that haloperidol abolished TNF-*α*-induced MMP-9 gene transcription through downregulation of NF-*κ*B activation in THP-1 cells.

### 3.6. Comparative Effects of Haloperidol on Downstream Signaling in Monocytic Gelatinolysis

**Figure 5:**
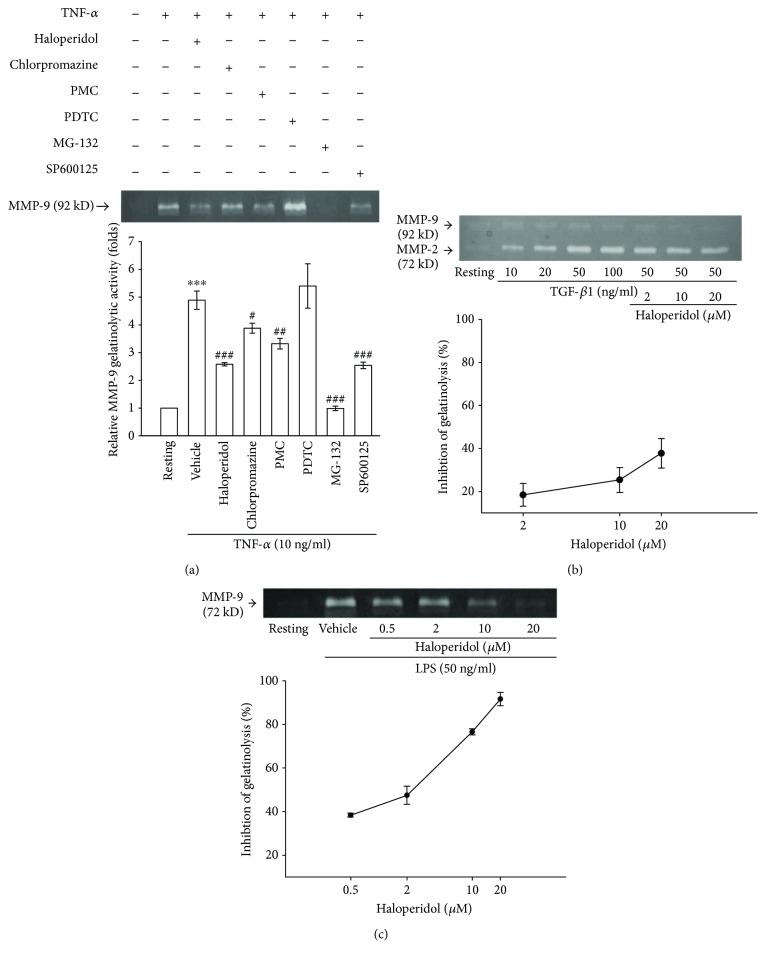
Comparative effects of haloperidol on downstream signaling in stimulated THP-1 cells. (a) THP-1 cells were pretreated with vehicle (DMSO), haloperidol (10 *μ*M), chlorpromazine (10 *μ*M), PMC (50 *μ*M), PDTC (100 *μ*M), MG-123 (1 *μ*M), or SP600125 (10 *μ*M) for 15 min before treatment with TNF-*α* (10 ng/ml) for 24 h. MMP-9-mediated gelatinolysis was then assayed by gelatin zymography. Bottom: densitometric analyses of gelatinolytic bands. Data are representative examples of three to five experiments. ^∗∗∗^*p* < 0.001, compared to the resting group; ^#^*p* < 0.05, ^##^*p* < 0.01, and ^###^*p* < 0.001, compared to the vehicle under stimulation. (b) THP-1 cells were pretreated with haloperidol (2, 10, and 20 *μ*M) or vehicle for 15 min before stimulation with transforming growth factor- (TGF-) *β*1. (c) THP-1 cells were pretreated with haloperidol (0.5, 2, 10, and 20 *μ*M) or vehicle for 15 min before stimulation with LPS. Cell-free conditioned medium was obtained and assayed for MMP-mediated gelatinolytic activity, as detailed in Methods. Percent inhibition (●) is presented as the mean ± SEM of three to five independent experiments.

Our studies showed that haloperidol reduced TNF-*α*-induced MMP-9 activation through the NF-*κ*B pathway (Figures 3 and 4). To unequivocally establish that the effect of haloperidol was unique and potent, we examined and compared its effects with different pharmacological agents and even used two different stimulators. As shown in Figure 5(a), haloperidol (10 *μ*M) strongly inhibited TNF-*α*-induced MMP-9 gelatinolysis by about 60% (lane 3). However, chlorpromazine, as a typical antidopaminergic and neuroleptic agent, caused only 24.4 ± 6.0% inhibition at the same concentration (lane 4). For the comparative studies of other NF-*κ*B inhibitors, this activation of gelatinolysis was significantly inhibited by about 38.8 ± 6.6% by PMC (50 *μ*M) but not by PDTC (100 *μ*M) (lanes 5 and 6). Moreover, MG-132 (1 *μ*M), as a proteasome inhibitor, and SP600125 (10 *μ*M), as a JNK inhibitor, also attenuated TNF-*α*-induced MMP-9 gelatinolysis by about 99.8 ± 2.3% and 59.1 ± 5.1%, respectively (lanes 7 and 8). However, MG-132 (1 *μ*M) markedly caused cellular viability to drop below 1% after treatment for 24 h (data not shown). We next examined the specific signaling action of haloperidol by different stimulators such as TGF-*β*1 and LPS. As shown in Figure 5(b), activation of THP-1 cells by TGF-*β*1 (50 ng/ml) induced a significant and submaximal increase in MMP-2- (72 kD) related gelatinolysis by 3.8 ± 0.2-fold compared to the resting control (lanes 1 and 4). After being pretreated with haloperidol (2, 10, and 20 *μ*M) for 15 min, MMP-2 activation by TGF-*β*1 was weakly affected (Figure 5(b), lanes 6–8). Even at a higher concentration of 20 *μ*M, haloperidol partially inhibited MMP-2 activation by about 37.7 ± 6.9% (Figure 5(b)). On the other hand, haloperidol (0.5, 2, 10, and 20 *μ*M) significantly and concentration-dependently inhibited LPS-stimulated MMP-9 gelatinolysis by 38.5 ± 0.9, 47.5 ± 4.2, 76.6 ± 1.4, and 91.6 ± 3.1%, respectively (Figure 5(c)). Its IC_50_ of gelatinolysis was about 3.1 ± 0.8 *μ*M. These results demonstrate that haloperidol selectively and markedly suppresses MMP-9 activation by LPS through the NF-*κ*B-related pathway but not MMP-2 activation by TGF-*β*1 through the Smad-related pathway in human monocytic cells.

### 3.7. The Effect of Haloperidol on Caudate/Putamen MMP-9 Activation by Microinjection of LPS in C57BL/6 Mice

**Figure 6:**
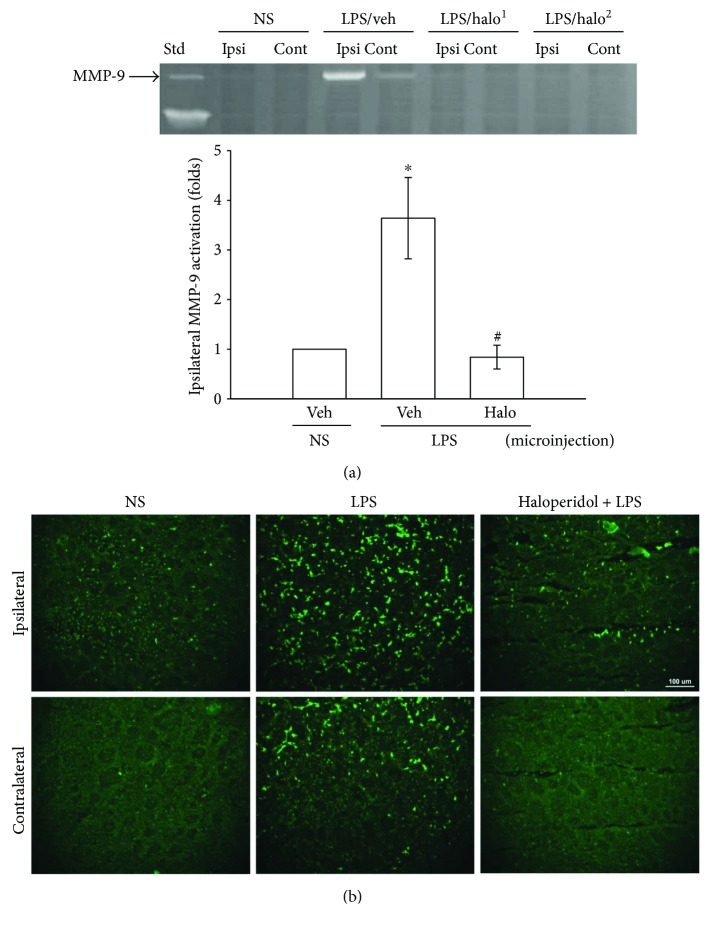
The effects of haloperidol on LPS-induced brain striatal gelatinolysis *in vivo*. Haloperidol (5 mg/kg) was administered intraperitoneally 1 h before and 25 min after LPS intrastriatal microinjection. (a) The representative gelatinolysis of MMP-9 activities in brain homogenates was assayed by methods as described. The samples of localized hemispheres (Ipsi: ipsilateral; Cont: contralateral) from normal saline-microinjected (NS), LPS with vehicle-treated (veh), and LPS with haloperidol-treated (halo^1^ and halo^2^) mice were shown with primarily MMP-9-mediated gelatinolytic activity. The leftmost lane was loaded with the culture media from HT1080 cells as a marker (Std) for MMP-2 and MMP-9. The relative folds of ipsilateral densitometric data were presented as the mean ± SEM. ^∗^*p* < 0.05 as compared to the vehicle plus NS-microinjected group; ^#^*p* < 0.05 as compared to the vehicle plus LPS-microinjected group. (b) Representative brain sections show in situ gelatinolytic activity within NS- or LPS-microinjected lesions in mice. The haloperidol-treated mice compared with vehicle-treated controls at 8 h after LPS microinjection. Data are representative example of four experiments. White scale bar indicates 100 *μ*m.

At 8 h after LPS microinjection in caudate/putamen region, the gelatinolytic activity in the ipsilateral (right) brain homogenate was significantly increased relative to its contralateral (left) or saline-microinjected ipsilateral brain homogenates in male C57BL/6 mice (Figure 6(a)). Surprisingly, the haloperidol- (5 mg/kg) treated group showed significantly decreased levels of ipsilateral pro-MMP-9 activity, as compared with the vehicle-treated group (Figure 6(a)). Next, in situ zymography confirmed the increased activity of MMP-9-mediated gelatinolysis in LPS-treated ipsilateral brain tissue, revealing a “spotty” localization of the gelatinase. Correspondingly, mice treated with haloperidol (5 mg/kg) also showed reduced gelatinolytic activity on ipsilateral tissue sections at 8 h after LPS microinjection as compared with the vehicle-treated control (Figure 6(b)).

## 4. Discussion

MMP activity is highly regulated by the crucial mechanisms [29]. In particular, expression of MMP-9 is inducible in the monocyte/macrophage lineage under stimulation of TNF-*α*, LPS, and proinflammatory cytokines [24, 30]. Overproduction of MMP-9 from these phagocytes and microglia has been implicated in the pathogenesis of several notable neurodegenerative diseases [31]. THP-1 cells are generally used as a good model for human monocyte/macrophage lineages and microglia. This human monocytic cell has already been used to induce production of proinflammatory cytokine and MMP-9 by amyloid *β* fibrils [32]. It is well known that TNF-*α* binds to TNF receptor type 1 (TNF-R1) to induce the production of MMP-9 in monocytic cells and monocytes [28]. According to our results, haloperidol significantly inhibited TNF-*α*-induced MMP-9-mediated gelatinolysis in a concentration-dependent manner. Its inhibition was not through interference with cellular viability or native MMP-9 catalytic activity. On the other hand, TIMP-1 as an endogenous inhibitor of MMP-9 has been reported to form an inactivated complex with pro-MMP-9 [33]. However, the content of TIMP-1 was not increased by haloperidol in activated THP-1 cells. Therefore, the inhibitory activity of haloperidol on MMP-9 gelatinolysis was not through a counterbalance with TIMP-1 content but proposed through downregulation of MMP-9 expression. Markedly, it was shown that haloperidol exerted its concentration-dependent inhibition of MMP-9 protein and m-RNA expression. From these points, it revealed that the inhibitory action of haloperidol was at the upstream of the transcriptional level.

The upstream signaling pathways of TNF-*α* that were utilized to stimulate MMP-9 production in monocytic cells were not fully understood. It was reported that TNF-*α* may activate receptor interacting protein 1 (RIP1) to induce NF-*κ*B activation, which is followed by initiation of p65/p50 transactivation and finally induction of MMP expression [34]. One study showed that TNFR-associated factor 2 (TRAF2) is sufficient to recruit the IKK (I*κ*B kinase) complex into the TNF-R1 signaling complex, whereas RIP is necessary for the activation of IKK [35]. As the classical NF-*κ*B pathway, TNF-*α* or LPS activates their receptors resulting in IKK activation and dissociation of phosphorylated I*κ*B*α* from the NF-*κ*B complex and then the proteolytic degradation of I*κ*B*α* by proteasomes, followed by translocation of activated p65/p50 (NF-*κ*B) into the nucleus and binding to relevant DNA responsive sites on the promoter region of the MMP-9 gene [36]. The present study showed that haloperidol suppressed I*κ*B*α* degradation and p65 transactivation in TNF-*α*-activated THP-1 cells. Furthermore, haloperidol also inhibited NF-*κ*B from binding to its cognate DNA element in the MMP-9 promoter by a ChIP assay. A related study also supported a major role of NF-*κ*B in MMP-9 production; the NF-*κ*B inhibitors significantly inhibit MMP-9 production induced by TNF-*α* or LPS in monocytes [30]. Thereafter, the anti-MMP-9 activation and gelatinolytic activities of classical NF-*κ*B inhibitors (PDTC, MG-132, and PMC) were also compared in this study. The results revealed that TNF-*α*-induced MMP-9 activation was significantly inhibited by MG-132 and PMC. Although PDTC is being used as a potent NF-*κ*B inhibitor in human monocytes [37], our results demonstrated that MMP-9 activation was paradoxically and slightly increased in PDTC-pretreated THP-1 cells. Consistent with our findings, previous reports showed that PDTC could activate JNK/SAPK in the macrophage cell line [38]. Our results also showed that MG-132 exerted a strong inhibition on TNF-*α*-induced MMP-9 activation and gelatinolysis, although cell death prominently occurred in THP-1 cells. It was reported that the NF-*κ*B-inhibitory property of MG-132 is dependent on its inhibition of the 26S proteasome [39]; however, the related inhibitors could induce proapoptotic effects in the monocytic cells [40]. Nevertheless, PMC as an *α*-tocopherol derivative with less cellular toxicity is an effective inhibitor of TNF-*α*-induced NF-*κ*B activation [41]. We found that haloperidol was more potent than PMC or PDTC in abrogating the activation of MMP-9. Taken together, it was revealed that haloperidol exerted the precise NF-*κ*B inhibition for reducing TNF-*α*-induced MMP-9 activation in the monocytic cells.

One of the major intracellular signal transduction pathways stimulated by TNF-*α* is the JNK MAPK pathway [42]. Pretreatment with a higher concentration of haloperidol partially inhibited JNK1 activation in THP-1 cells. This result is consistent with prior work by Brenner et al. [43] who suggested that JNK is involved in signal transduction stimulated by TNF-*α* on the induction of collagenase. It was also consistent with our inhibitory results of SP600125 to support an important role of JNK activation in TNF-*α*-induced MMP-9 activation in THP-1 cells. In particular, TRAF2 is essential for TNF-*α*-mediated JNK activation but not for NF-*κ*B activation [35]. On the other hand, the deletion of the gene encoding RIP inhibited TNF-*α*-induced NF-*κ*B activation but had a minimal effect on JNK activation [44]. Therefore, there are convincing data to suggest that JNK is, at least partially, a potential transcriptional element in MMP-9 induction by TNF-*α* in THP-1 cells, although haloperidol had been found to induce the expression of dual-specificity phosphatase 6 [45]. However, the basis of the inhibition on the JNK1 activation by high concentration of haloperidol was not clearly known. According to our results of less inhibition of the JNK pathway, it was proposed that haloperidol may inhibit the RIP/IKK pathway, but this requires further investigation. On the other hand, haloperidol was shown to significantly stimulate pERK1 MAPK expression at the striatum *in vivo* [46]. Also, the phosphorylation of MEK-ERK-p90RSK was upregulated by haloperidol via protein phosphatase 2A [20]. Therefore, it was proposed that haloperidol would not inhibit but rather increase ERK MAPK activation.

As a Toll-like receptor- (TLR-) 4 activator, lipopolysaccharide (LPS) causes the stimulation of monocytes to induce MMP-9 expression through an NF-*κ*B pathway [47]. Alternatively, the specific event precedes the different expression of MMP-2 after stimulation of monocytic cells by TGF-*β*. The Smad pathway is activated to induce MMP-2 production in response to TGF-*β* [48]. In this comparative study of different stimulators, we showed that haloperidol exerted more inhibition on LPS-induced MMP-9 than TGF-*β*-induced MMP-2 activation in the monocytic cells. Therefore, the inhibitory mechanisms of haloperidol could be selectively mediated by suppression of the NF-*κ*B pathway, resulting in the abrogation of MMP-9 expression and activation.

It is very important to determine whether the concentrations of haloperidol used in this study are clinically relevant and achievable. It was shown that concentrations of haloperidol in brain tissues are 10- to 30-folds higher than clinically relevant serum concentrations of 0.1 to 1 *μ*M in the treatment of schizophrenia [49, 50], although the exact concentrations of haloperidol in different brain areas are not clearly known. However, the present results suggest that dosage of haloperidol is most likely to exert its inhibition on MMP-9 activation in the micromolar range among brain tissues in therapeutic conditions. On the other hand, chlorpromazine, a classical neuroleptic agent with anti-inflammatory properties [51], is less potent than haloperidol at inhibiting TNF-*α*-induced MMP-9 activation in THP-1 cells. In accordance with few dopamine receptors being expressed on human monocytes [52], it is proposed that the inhibitory mechanism of haloperidol on MMP-9 expression is not mediated by a dopaminergic receptor blockade.

Much evidence has shown that LPS and the danger-associated molecular patterns are major contributors in neuroinflammation and neurotoxicity through TLR-4 activation during brain injury [53]. Also, TLR-4 was proposed as an important therapeutic target for stroke-induced neuroinflammation [54]. Many clinical and experimental studies have provided converging evidence of the central role of MMP-9 in the pathogenesis of inflammatory brain disorders [55]. The manipulation of LPS treatment could induce neuroinflammation and neurological symptoms similar to those of neurodegenerative diseases [56]. As expected that the marked enhancement of MMP-9 activity was noted in the brain region of intracerebral LPS-microinjected B6 mice when compared with saline-microinjected mice, these results were consistent with the findings of an earlier investigation [57]. The present study clearly indicated that haloperidol exhibited a significant reduction of the MMP-9 activation in the striatum region during LPS-induced neuroinflammation *in vivo*. Thus, in alignment with a previous manipulated animal study, the NF-*κ*B inhibition significantly attenuated cerebral MMP-9 activity in brain inflammation [58].

## 5. Conclusions

Taken together, haloperidol showed a strong inhibition on the activation and expression of MMP-9 by TNF-*α* stimulation in the human monocytic cells. The inhibitory mechanism of haloperidol may be due to its reversal of I*κ*B*α* properties followed by inhibition of NF-*κ*B transactivation, with a partial influence on JNK, and subsequent inhibition of MMP-9 expression. The suppression of MMP-9 expression and activation by haloperidol *in vitro* and *in vivo* may explain its crucial role as a bystander in antineuroinflammatory actions for the treatment of psychotic disorders.
